# ATTITUDES TOWARD PARENTAL FINANCIAL SUPPORT OF YOUNG-ADULT CHILDREN

**DOI:** 10.1093/geroni/igz038.2489

**Published:** 2019-11-08

**Authors:** Radion Svynarenko

**Affiliations:** University of Kentucky, Lexington, Kentucky, United States

## Abstract

Studies have shown that Americans do not save enough for retirement because they prioritize providing support of their young-adult children over saving for retirement. Attitudes toward parental support has been largely overlooked in existing literature. Using a factorial vignette experimental design and a sample of 500 Americans of age 40 and older, this study investigated how manipulation of contextual factors changed endorsement of parental support. This study found that endorsement of parental support varied depending on the goal of support, whether it was to purchase a car, pay for school tuition, or to pay for down payment for a house. Thus, 67% of respondents endorsed parental financial assistance with purchasing a car, 44% endorsed down payment for a house, and only 38% endorsed paying for college tuition, reflecting overall social importance of these three elements in becoming an adult person. Gender of the child did not affect endorsement of parental financial support to adult children, indicating that there were no gender specific social expectations of who should receive more support from parents, daughters or sons. The major motives of parental support included (a) desire to be a “good parent” and to take responsibility for the child, (b) expectation that children would eventually pay back their parents, and (c) desire to make sacrifice for own children. Parental support may provide numerous benefits to both children and their parents; however, it is important to educate parents on ways to support their children without threatening their own financial needs in retirement.

